# Generative Artificial Intelligence to Transform Inpatient Discharge Summaries to Patient-Friendly Language and Format

**DOI:** 10.1001/jamanetworkopen.2024.0357

**Published:** 2024-03-11

**Authors:** Jonah Zaretsky, Jeong Min Kim, Samuel Baskharoun, Yunan Zhao, Jonathan Austrian, Yindalon Aphinyanaphongs, Ravi Gupta, Saul B. Blecker, Jonah Feldman

**Affiliations:** 1 Division of Hospital Medicine, Department of Medicine, NYU (New York University) Langone Health, New York, New York; 2 Department of Medicine, NYU Long Island School of Medicine, Mineola; 3 Department of Population Health, NYU Langone Health, New York; 4 Department of Health Informatics, NYU Langone Medical Center Information Technology, New York; 5 Predictive Analytics Unit, NYU Langone Health, New York; 6 Department of Internal Medicine, Long Island Community Hospital, NYU Langone Health, New York

## Abstract

**Importance:**

By law, patients have immediate access to discharge notes in their medical records. Technical language and abbreviations make notes difficult to read and understand for a typical patient. Large language models (LLMs [eg, GPT-4]) have the potential to transform these notes into patient-friendly language and format.

**Objective:**

To determine whether an LLM can transform discharge summaries into a format that is more readable and understandable.

**Design, Setting, and Participants:**

This cross-sectional study evaluated a sample of the discharge summaries of adult patients discharged from the General Internal Medicine service at NYU (New York University) Langone Health from June 1 to 30, 2023. Patients discharged as deceased were excluded. All discharge summaries were processed by the LLM between July 26 and August 5, 2023.

**Interventions:**

A secure Health Insurance Portability and Accountability Act–compliant platform, Microsoft Azure OpenAI, was used to transform these discharge summaries into a patient-friendly format between July 26 and August 5, 2023.

**Main Outcomes and Measures:**

Outcomes included readability as measured by Flesch-Kincaid Grade Level and understandability using Patient Education Materials Assessment Tool (PEMAT) scores. Readability and understandability of the original discharge summaries were compared with the transformed, patient-friendly discharge summaries created through the LLM. As balancing metrics, accuracy and completeness of the patient-friendly version were measured.

**Results:**

Discharge summaries of 50 patients (31 female [62.0%] and 19 male [38.0%]) were included. The median patient age was 65.5 (IQR, 59.0-77.5) years. Mean (SD) Flesch-Kincaid Grade Level was significantly lower in the patient-friendly discharge summaries (6.2 [0.5] vs 11.0 [1.5]; *P* < .001). PEMAT understandability scores were significantly higher for patient-friendly discharge summaries (81% vs 13%; *P* < .001). Two physicians reviewed each patient-friendly discharge summary for accuracy on a 6-point scale, with 54 of 100 reviews (54.0%) giving the best possible rating of 6. Summaries were rated entirely complete in 56 reviews (56.0%). Eighteen reviews noted safety concerns, mostly involving omissions, but also several inaccurate statements (termed hallucinations).

**Conclusions and Relevance:**

The findings of this cross-sectional study of 50 discharge summaries suggest that LLMs can be used to translate discharge summaries into patient-friendly language and formats that are significantly more readable and understandable than discharge summaries as they appear in electronic health records. However, implementation will require improvements in accuracy, completeness, and safety. Given the safety concerns, initial implementation will require physician review.

## Key Points

**Question:**

Can a large language model transform discharge summaries into a format that is more readable and understandable for patients?

**Findings:**

In this cross-sectional study of 50 discharge summaries, understandability scores were significantly higher for patient-friendly discharge summaries. Summaries were rated entirely complete in 56 of 100 reviews, but 18 reviews noted safety concerns involving omissions and inaccuracies.

**Meaning:**

These findings suggest that a large language model could be used to translate discharge summaries into patient-friendly language and format, but implementation will require improvements in accuracy, completeness, and safety.

## Teaser

This cross-sectional study measures the readability and understandability of artificial intelligence (AI)-generated patient-friendly discharge summaries compared with the original discharge summaries and tests the accuracy and completeness of the patient-friendly version.

## Introduction

Actively involving patients in their own care improves their health-related outcomes.^1,2^ Implementation of the 21st Century Cures Act mandates patients’ access to their clinical notes and other information in their electronic health records.^3^ Increased patient access to their clinical notes through widespread availability of electronic patient portals^4^ has the potential to improve patient involvement in their own care, as well as confidence in their care from their care partners.^5,6,7^ However, clinical notes are typically filled with technical language and abbreviations that make notes difficult to read and understand for patients and their care partners. This issue can create unnecessary anxiety or potentially delay care recommendations or follow-up for patients and their families. Generative artificial intelligence (AI) has the potential to transform medical information into a patient-friendly language and format that supports high-quality care.^8^

Discharge from inpatient acute care medicine units is a complex process that has been a frequent focus of transition of care interventions. One important aspect of successful transition from inpatient to outpatient care includes educating patients about their own health.^9^ Studies have shown poor quality of written discharge information, with up to 88% of discharge instructions not readable to the population served.^10,11,12,13^ Meanwhile, studies suggest that improving readability in discharge summaries might improve outcomes like readmissions.^14^ Some efforts to remedy this disconnect include developing patient-friendly discharge letters^15^ or using student volunteers to help patients understand their notes.^16^ Early studies have already shown that AI-generated plain language notes may be more usable, beneficial to the patient-clinician relationship, and empowering to patients.^17^

Generative AI has become a potential disruptor across many fields, including medicine.^18^ Research on generative AI products in a medical context has shown that they can pass standardized United States Medical Licensing Examinations^19^ and write clinical notes.^20^ Generative AI has also shown promise in creating medical materials meant for patient consumption. For example, in responses to medical questions, generative AI has given high-quality and empathic responses.^19,21^ Studies have also shown that generative AI may be useful for creating lay summaries of research articles.^22^

However, despite its promise, implementing generative AI technologies in patient care has raised important patient safety and physician liability concerns.^23^ Inaccuracies in generative AI content are a major source of this concern. These inaccurate albeit convincing outputs are termed *hallucinations*. For instance, Lee et al^18^ highlighted an AI chatbot that generated a medical summary from a dialogue between a patient and clinician. The summary indicated that the patient’s body mass index was 14.8 when in fact the necessary information to calculate it was not present.^18^ It is notable that when the chatbot was asked to “reread” the output, it was able to identify the hallucination.

No published work to our knowledge has investigated the use of current-generation generative AI platforms to transform real medical notes into patient-friendly language and format. In our study, we hypothesized that with appropriate programming, a large language model (LLM) could create safe and accurate patient-friendly discharge summaries. To test this hypothesis, we measured the readability and understandability of these patient-friendly discharge summaries and compared them with the readability and understandability of the original discharge summaries. As balancing metrics, we tested accuracy and completeness of the patient-friendly discharge summary.

## Methods

This cross-sectional study, as part of a larger project to improve care delivery in our health system, was deemed exempt from institutional review board review based on the NYU Langone Health self-certification protocol. The study followed the Strengthening the Reporting of Observational Studies in Epidemiology (STROBE) reporting guideline.

This was a cross-sectional review of 50 inpatient discharge summaries. The number 50 was chosen a priori based on feasibility. We used Epic Systems reporting workbench to export a dataset containing metadata for all notes of the Discharge Summary Note type across NYU Langone Health Systems from June 1 to 30, 2023, totaling 5025 summaries. We used the Excel 2016 rand() function (Microsoft Corporation) to generate a random number corresponding to each note and selected the 200 notes with the lowest random number. A single reviewer confirmed the identified notes were actual discharge summaries written by the General Internal Medicine service and that the patients were not discharged as dead. For final inclusion in the study, we selected 50 of the remaining notes with the lowest random numbers. Our sample included discharges from all of NYU Langone’s hospital campuses and did not include more than 1 discharge from any single patient. Discharge summaries as they appear in the medical record, which we refer to as original discharge summaries, were used as inputs in our generative AI prompt, and the outputs were what we refer to as patient-friendly discharge summaries.

**Figure 1.**
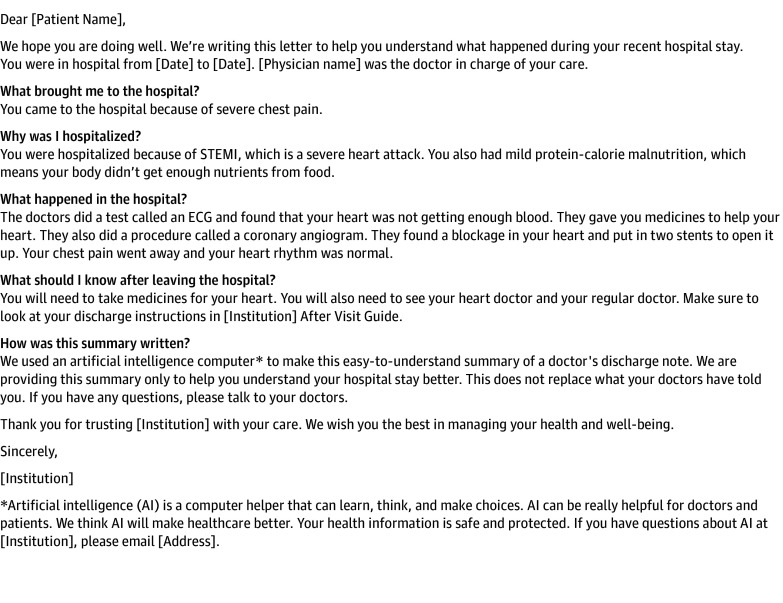
Example of a Patient-Friendly Discharge Summary Created Using a Large Language Model This example was created from a hypothetical discharge summary. ECG indicates electrocardiogram; STEMI, ST-segment elevation myocardial infarction.

Our project team first worked with the organization’s patient family council to understand characteristics that made a discharge summary patient friendly. We then reviewed our institution’s discharge summary standard and reviewed scholarly sources. Using this information, we identified a subset of required elements that we aimed to include in the patient-friendly summary: admission date, discharge date, indication for admission, history of present illness, hospital course, diagnoses, procedures, and discharge physician. Elements of the original discharge summary that we aimed to exclude from the patient friendly version were the billing and coding table, admission source, diagnostic studies, admitting physician, and discharge condition. In line with the best practice for patient education materials, we made the patient-friendly summary 1 page in length. We chose a question-and-answer format. Figure 1 presents an example of a patient-friendly discharge summary format.

### Statistical Analysis

We developed the prompt using the institutionally licensed, Health Insurance Portability and Accountability Act–compliant version of GPT-4 on the Azure Open AI studio platform. The prompt development team included a physician data scientist, 2 board-certified physician informaticists, 2 board-certified internal medicine specialists, a resident physician, and an AI engineer (J.Z., J.M.K., S.B., J.A., Y.A., and J.F.). Working independently, physician team members began with basic prompts testing and refining, evolving their approach through hundreds of iterations, a process known as prompt engineering. The physicians individually kept experiment journals, documenting test results for each iteration, and met weekly for 6 weeks comparing findings with each other and refining their approach. Since much of the prompt design came from several members of our group working independently, we used the following directive to align our efforts: “create a discharge summary for a hospitalization on acute care medicine unit that is readable for a patient at a sixth grade reading level.” At the end of the 6 weeks, the best-performing prompt was chosen. This prompt used few-shot learning, a technique where the AI system learns by example from sample inputs and outputs. To see our prompt, refer to eFigure 1 in Supplement 1. We did not include the original discharge summaries used for prompt engineering in the final study. The process of prompt creation was undertaken from May 24 to July 13, 2023.

The 50 original discharge summaries were preprocessed to include only elements of the aforementioned discharge summary. Then they were input into the LLM together with the engineered prompt to generate 50 patient-friendly discharge summaries. All discharge summaries were processed by the LLM between July 26 and August 5, 2023. For evaluating readability, we used the Flesch-Kincaid readability tests. These include the Flesch-Kincaid Reading Ease score and Flesch-Kincaid Reading Grade Level, both of which use word and sentence lengths to estimate how difficult an English text is to understand.^24^ We used the total word count as a measure of document length. We used Microsoft Word software to measure Flesch-Kincaid scores and total word count.^25,26^

To assess understandability, we used the Patient Education Materials Assessment Tool (PEMAT) understandability scale. The PEMAT is a validated instrument specifically designed to gauge the extent to which patients can comprehend and act on health-related educational resources.^27,28^ The PEMAT consists of 2 independent scales: one scoring materials based on 19 understandability criteria, and another on a 7-point actionability scale.^27^

The understandability scale focuses on 19 aspects related to clarity, organization, layout, and the use of visual aids. Each element is rated as 0 (does not satisfy the criteria), 1 (meets the criteria), or not applicable. The final score is calculated as a percentage, with higher scores indicating superior understandability. As our organization’s discharge summaries do not include patient actions, which are instead provided in the discharge instructions, the actionability scale component of PEMAT was not included in our analysis.

We used 2 independent physician reviewers (J.Z. and J.M.K.) to evaluate the understandability of all 50 patient summaries using the 19-point understandability scale. Where statistical testing is performed, the results are taken as the mean of the scores given by the 2 reviewers.

We separately recruited 25 resident physician volunteers to evaluate accuracy and completeness of the patient-friendly discharge summaries. These physicians regularly compose discharge summaries and are familiar with the standard elements of discharge summaries at our institution. Each discharge summary was reviewed by 2 of the resident physicians independently. Accuracy was measured on a 6-point scale modeled after previous accuracy scales developed for generative AI research.^29^ The original discharge summary was used as the criterion standard judgment of accuracy. We then further reviewed free-text description of inaccuracies for patterns to see if categories emerged. For reviews that reported any inaccuracy, we asked whether there was any potential safety concern. For completeness, we asked reviewers to indicate whether each of the prespecified 8 elements of the discharge summary was present (eFigure 2 in Supplement 1). We considered the possibility that the simplified summary could contain useful content that was not included in the criterion standard note. We did not measure this scenario.

We used paired *t* tests to compare the differences in word count, Flesch-Kincaid Reading Ease score, and Flesch-Kincaid Reading Grade Level between original and patient-friendly discharge summaries. Since the PEMAT score is not normally distributed, we used the Wilcoxon rank sum test to examine the association with the 2 groups. Additionally, we conducted an analysis of variance to access the association of sex and race and ethnicity reported in the electronic medical record (owing to concerns regarding gender and racial biases in AI-based technologies), length of stay, and original discharge summary word count with accuracy. Interrater reliability was calculated using the percentage agreement. All statistical analyses were performed using R, version 4.3.0 (R Project for Statistical Computing). Two-sided *P* < .05 was considered statistically significant.

## Results

**Figure 2.**
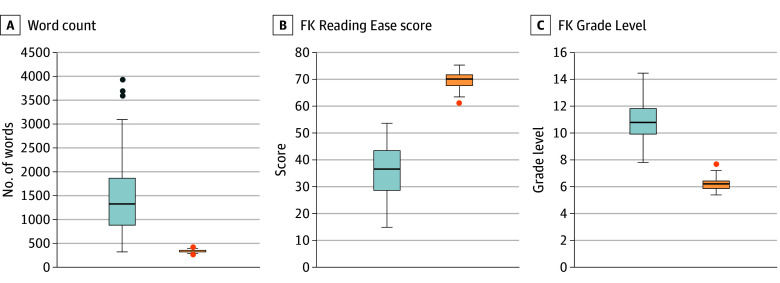
Readability of Discharge Summaries Box plot compares total word count, Flesch-Kincaid (FK) Reading Ease score, and FK Grade Level. Blue boxes represent the original discharge summary; orange boxes represent the patient-friendly discharge summary. For FK Reading Ease score, higher scores indicate better outcomes; for FK Grade Level, lower grade levels indicate better outcomes. Vertical lines indicate means; box, IQRs; dots, outliers; and error bars, minimum and maximum excluding outliers.

Among 50 patient discharges included in the analysis, 31 patients (62.0%) identified as female and 19 (38.0%) identified as male, with a median age of 65.5 (IQR, 59.0-77.5) years. Racial and ethnic identity in the electronic health record included 2 (4.0%) Asian patients, 15 (30.0%) Black or African American patients, 25 (50.0%) White patients, and 8 (16.0%) patients of other race or ethnicity (including Filipino; Hispanic, Latino, or Spanish; multiracial Black and White; and not listed) (Table 1). We compared measures of readability between the original and the patient-friendly discharge summaries (Figure 2). We found the patient-friendly discharge summaries had fewer words than the original discharge summary, a difference that was statistically significant (mean [SD], 338 [256] vs 1520 [904] words; *P* < .001). Readability as measured by the Flesch-Kincaid Reading Ease score was significantly higher in the patient-friendly discharge summaries compared with the original discharge summaries (mean [SD], 69.5 [3.2] vs 35.5 [9.8]; *P* < .001). Conversely, Flesch-Kincaid Grade Level was significantly improved (ie, was lower) for patient-friendly discharge summaries (mean [SD], 6.2 [0.5] vs 11.0 [1.5] grade levels; *P* < .001)

**Table 1.** Patient Characteristics^a^

Characteristic	Values (N = 50)
Age, median (IQR), y	65.5 (59-77.5)
Sex	
Female	31 (62.0)
Male	19 (38.0)
Race	
Asian	2 (4.0)
Black or African American	15 (30.0)
White	25 (50.0)
Other^b^	8 (16.0)
Length of stay, median (IQR), d	5 (3-8)
Discharge disposition	
Home or self-care	21 (42.0)
Home under care of home health services	17 (34.0)
Skilled nursing facility	8 (16.0)
Other	4 (8.0)

^a^ Unless otherwise indicated, data are expressed as No. (%) of patients.

^b^ Includes electronic health record entries Filipino; Hispanic, Latino, or Spanish; multiracial Black or African American and White; and not listed.

We found a significantly improved understandability based on PEMAT in the patient-friendly discharge summaries, with a score of 81% compared with 13% in original discharge summaries (*P* < .001). Interrater reliability was good, with 97% agreement.

**Figure 3.**
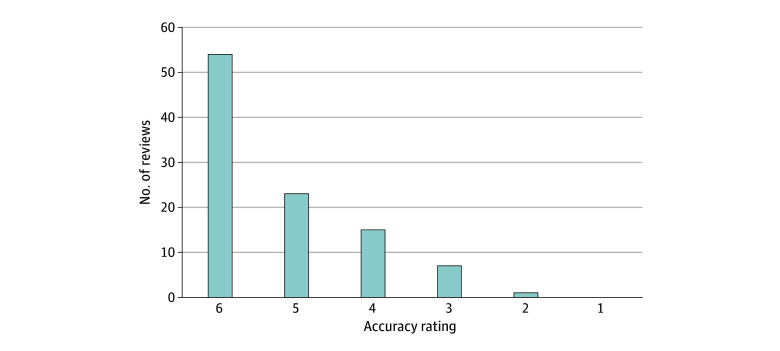
Counts of Accuracy Ratings The counts of accuracy ratings on a scale of 1 to 6 from each of 100 reviewers, where 1 indicates completely incorrect; 2, more incorrect than correct; 3, approximately equally correct and incorrect; 4, more correct than incorrect; 5, nearly all correct; and 6, completely correct.

With regard to accuracy, 54 of 100 reviews gave a top box rating (Figure 3). None of the reviews rated accuracy as completely incorrect, and only 1 of the 100 was rated as more incorrect than correct. Interrater reliability of top box accuracy was poor at 48%. For the reviews that were not given the top box rating of accuracy, 18 of 46 (39.1%) were considered to have potential safety risks. Free-text descriptions of inaccuracy were found to fall into 3 categories after our review: omissions, hallucinations, and other. The most common inaccuracy was omission, making up 24 of 46 (52.1%), while hallucinations made up 4 of 46 (8.7%), and the rest fell into the other category (18 of 46 [39.1%]). For examples of inaccuracies, see Table 2.

**Table 2.** Reviewer Comments on Inaccuracies Found in Patient-Friendly Discharge Summaries^a^

Category of inaccuracy	Safety risk	Reviewer comment
Hallucination	No	The patient-friendly discharge summary stated dizziness got better, but there is no mention in the original discharge narrative that this improved.
Hallucination	No	Some abbreviations are incorrect, and some numbers were incorrect for laboratory work.
Hallucination	No	Though the patient-friendly discharge summary notes infection, there was no indication of infection according to the original discharge summary.
Hallucination	Yes	The answer to “What brought me to the hospital” is wrong. The patient-friendly discharge summary states “because of chest pain.” Chest pain is nowhere mentioned in hospital course.
Omission	Yes	The patient-friendly discharge summary did not mention nausea and vomiting as part of why they came to hospital. It also did not mention 2 nonspecific findings on imaging.
Other	Yes	The patient-friendly discharge summary mentioned that they “gave water pills for heart problems” and listed one of the problems in the hospitalization as “heart problems,” which may sound like the patient had a heart failure exacerbation when instead they were just resuming their home medications.

^a^ Here we include all 4 comments categorized as hallucination as well as 1 example from each of the other 2 categories. Comments by reviewers are lightly edited for clarity and patient privacy.

As for completeness, 56 of the 100 reviews rated as entirely complete, 26 had only 1 category incomplete, 11 had 2 categories incomplete, 6 had 3 categories incomplete, and 1 had 4 categories incomplete (eFigure 3 in Supplement 1). The most common category graded as incomplete was procedures. Of the 43 reviews that indicated a procedure was performed, 17 (39.5%) were graded as incomplete. The second most common category graded as incomplete was history of present illness, with 25 of 100 (25.0%) being incomplete. The next most common was procedure, with 17 of 100 reviews indicating that this was incomplete. Interrater reliability was good across categories at 88%.

We did not find any association between word count in the original discharge summary and accuracy nor between length of stay and accuracy. Given the presence of bias reported in AI,^30^ we explored whether accuracy was associated with race or sex and did not find such an association (eTable in Supplement 1).

## Discussion

In this cross-sectional study of 50 discharge summaries, we found that generative AI successfully transformed discharge summaries into a format that was more readable and understandable for patients. Conventional standards of readability recommend creating materials appropriate for a sixth grade reading level.^31^ We were able to show that our patient-friendly discharge summaries were consistently at a sixth or seventh grade reading level. This is markedly different from original discharge summaries, in which reading ease varies widely but is typically at the 11th grade reading level. We measured understandability using the PEMAT instrument and found patient-friendly discharge summaries to have high levels of understandability. This is also a marked difference from original discharge summaries.

It is notable that in regard to word count, we found a marked difference, with original discharge summaries having a high mean word count of 1520. While we did not parse out why these word counts were high, we suspect that unnecessarily duplicated material makes up a large portion, which has been suggested in the literature.^32^ These high word counts may be a reason that patient records are inaccessible and illustrate where generative AI can be helpful in sorting through large amounts of information.

While others have used various AI models to develop discharge summaries,^33^ to our knowledge, our effort represents the first use of new-generation AI platforms on real patients’ notes to transform them into a patient-friendly format. This also accords with the mission of certain patient advocacy groups, such as OpenNotes,^34^ to empower patients to own and understand their own health information and increase transparency.

Despite this success, our balancing measures of accuracy and completeness need to be addressed before any future deployment. Only 54 of 100 reviews had top box accuracy, and safety concerns were reported in 18. While previous studies have highlighted bias and hallucination as a cause of generative AI inaccuracies, most inaccuracies in our study were attributed to omission of key information (52.1%) vs hallucination (8.7%).^35^ Since all omitted information must be contained in the original discharge summary that is made available to patients, we think that safety concerns due to omissions will be of less risk during deployment. Our bigger worry consisted of inaccuracies due to hallucinations. It is also worth noting we did not find an association between accuracy and either sex or race to suggest bias, though it is important to keep in mind that our study was not powered for this question. In regard to completeness, we found that components most frequently missing from the patient-friendly discharge summaries were history of present illness and procedures.

We think a major source of inaccuracy due to omission and incompleteness comes from prompt engineering that optimized for readability and understandability. For example, limiting the number of words in a sentence or a document is considered more understandable. This makes it difficult to provide a detailed, comprehensive description of a complex patient condition. Future iterations will have to explore the trade-off between readability and understandability on one hand and completeness on the other.

### Limitations

Our study has several limitations. First, we limited our patient-friendly discharge summaries to English. This is a major issue with original discharge summaries as well and is a barrier to transparent, equitable care of non–English-speaking patients and caregivers.^36,37^ We hope to include non-English outputs in future iterations of our initiative. Another limitation was validation of survey instruments. Given the novelty of using generative AI on medical records, there is a paucity of validated instruments to measure accuracy and completeness. We developed an original instrument to do this; therefore, it does not have robust validation. Furthermore, while PEMAT is well validated for patient educational materials, it has not been validated in this particular use. Interrater reliability was good for PEMAT and completeness measurements; however, accuracy showed low levels of interrater reliability. To further test the significance of our survey results, future directions might include using human-generated patient-friendly discharge summaries as a comparator group for accuracy and completeness.

Another limitation is the lack of generalizability. We looked at only 50 discharge summaries from general medicine floors during a single month in 1 health system, so our results may not be generalizable to other settings. Furthermore, the prompt was specifically designed for use within our health care system.

## Conclusions

In this cross-sectional study, the strong performance of our generative AI prompt in creating highly readable and understandable discharge summaries shows the promise of using generative AI to make patient information more accessible to patients themselves. However, limitations in accuracy and completeness, which in turn affect safety, remain a hurdle for full-scale implementation. To address these limitations, we will further explore prompt strategies such as chain of thought and use of additional inputs from the patient records, such as progress notes. Initial implementation will require robust clinical safeguards such as physician review before publishing the output to patients.

## Notes

Supplement 1. **eFigure 1.** Artificial Intelligence–Generated Prompt Used for Our Study **eFigure 2.** Accuracy and Completeness Survey **eFigure 3.** Counts of Incomplete Elements in the Patient-Friendly Discharge Summary **eTable.** Between-Group Differences in Accuracy: ANOVA

Supplement 2.
Data Sharing Statement
