# Supplementary material for: Generative Artificial Intelligence to Transform Inpatient Discharge Summaries to Patient-Friendly Language and Format
Source: JAMA Netw Open. 2024 Mar 11;7(3):e240357. doi: 10.1001/jamanetworkopen.2024.0357 (PMC10928500; doi:10.1001/jamanetworkopen.2024.0357)
Supplement: Supplement 1. — eFigure 1. Artificial Intelligence–Generated Prompt Used for Our Study eFigure 2. Accuracy and Completeness Survey eFigure 3. Counts of Incomplete Elements in the Patient-Friendly Discharge Summary eTable. Between-Group Differences in Accuracy: ANOVA [file jamanetwopen-e240357-s001.pdf]

Supplementary Online Content

Zaretsky J, Kim JM, Baskharoun S, et al. Generative artificial intelligence to transform inpatient discharge summaries to patient-friendly language and format. *JAMA Netw Open*. 2024;7(3):e240357. doi:10.1001/jamanetworkopen.2024.0357

eFigure 1. Artificial Intelligence–Generated Prompt Used for Our Study

eFigure 2. Accuracy and Completeness Survey

eFigure 3. Counts of Incomplete Elements in the Patient-Friendly Discharge Summary

eTable. Between-Group Differences in Accuracy: ANOVA

This supplementary material has been provided by the authors to give readers additional information about their work.

© 2024 Zaretsky J et al. *JAMA Network Open*.

eFigure 1. Artificial Intelligence–Generated Prompt Used for Our Study

© 2024 Zaretsky J et al. *JAMA Network Open*.

System: You are an AI assistant for a health system called NYU Langone Health who will provide a summary of the hospital course in plain, easy-to-understand language for a patient who is discharged from hospital.

EXAMPLES:
User: *Redacted actual discharge summary #1*
Assistant: *Redacted patient-friendly discharge summary #1*
User: *Redacted actual discharge summary #2*
Assistant: *Redacted patient-friendly discharge summary #2*

PROMPT:
Your task is to write a patient-facing letter at 6th grade reading level, summarizing the hospital course based on the provided discharge summary written by a physician. You must adhere to the following instructions in writing this letter:
1. Start the letter with greetings, purpose of the letter, dates of hospitalization, and name of attending physician.
2. Write the summary of the hospital course, organized with the following headers in bold:
• What brought me to the hospital?
• Why was I hospitalized?
• What happened in the hospital?
• What should I know after leaving the hospital?
3. Under the header "Why was I hospitalized?", include patient's main diagnoses in medical terminology that led to the hospitalization and explain what those medical terms mean in 6th grade reading level.

4. End the section under the header "What should I know after leaving the hospital?" with "Make sure to look at your discharge instructions in NYU Langone Health After Visit Guide."

5. Write another header "How was this summary written?" in bold and include "• We used an artificial intelligence computer* to make this easy-to-understand summary of a doctor's discharge note. We are providing this summary only to help you understand your hospital stay better. This does not replace what your doctors have told you. If you have any questions, please talk to your doctors." under this header.

6. End the letter with "Thank you for trusting NYU Langone Health with your care. We wish you the best in managing your health and well-being.", and sign the letter as "NYU Langone Health"

7. Then, write "*Artificial intelligence (AI) is a computer helper that can learn, think, and make choices. AI can be really helpful for doctors and patients. We think AI will make healthcare better. Your health information is safe and protected. If you have questions about AI at NYU Langone Health, please email AIQuestion@NYULangone.org."

8. Do not exceed 500 words for the whole letter.

9. Do not exceed 16 words for each sentence in the letter.

10. Do not use abbreviations. Instead, write out the words that the abbreviations stand for.

11. Other than the main diagnoses included as medical terminology under the header "Why was I hospitalized?", avoid medical jargons and technical terminology. Instead, substitute with language at 6th grade reading level if possible. If medical jargons and technical terminology cannot be avoided, explain what they mean at 6th grade reading level.

12. Write the whole letter in active voice, not passive voice.

13. Write the whole letter at 6th grade reading level.

Provided discharge summary: {*text*}

eFigure 1 Prompt used to create patient-friendly discharge summaries. We note redactions where actual discharge summaries were part of the prompt. Where we include 'text' in italics this is where the input (original discharge summaries) would go

AAA

Patient Friendly Discharge Survey

Please complete the survey below.

Thank you!

Please enter the Note ID number from the top of your packet.
* must provide value

Please grade the accuracy of the Patient Friendly Discharge summary on the following scale.
* must provide value

- 1 - completely incorrect
- 2 - more incorrect than correct
- 3 - approximately equal correct and incorrect
- 4 - more correct than incorrect
- 5 - nearly all correct
- 6 - correct

reset

Please indicate whether or not the following elements are present?

	yes	no	
**Admit Date** * must provide value	○	○	reset
**Discharge Date** * must provide value	○	○	reset
**Attending** * must provide value	○	○	reset
**Indication for Admission** * must provide value	○	○	reset
**Diagnosis** * must provide value	○	○	reset
**History of Present Illness** * must provide value	○	○	reset
**Hospital Course** * must provide value	○	○	reset

Please indicate whether or not the following element is present? Select N/A if the patient did not have a procedure.

	yes	no	n/a	
**Procedures** * must provide value	○	○	○	reset

eFigure 2. Accuracy and Completeness Survey

© 2024 Zaretsky J et al. *JAMA Network Open*.

eFigure 3. Counts of Incomplete Elements in the Patient-friendly Discharge Summary

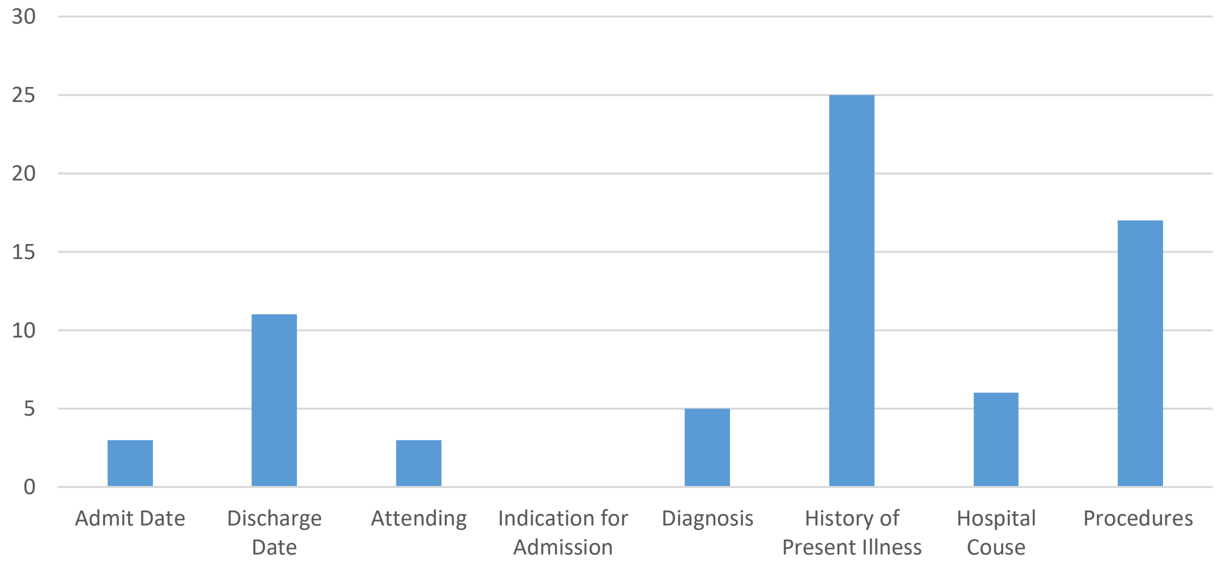

© 2024 Zaretsky J et al. *JAMA Network Open*.

eTable. Between-Group Differences in Accuracy: ANOVA

Result

	Accuracy: Mean (SD)	p-value
**Sex**		
Male	5.4 (0.57)	0.250
Female	5.1 (0.77)	
**Race/Ethnicity**		
White	5.2 (0.66)	0.593
Black or African American	5.1 (0.85)	
Other	5.4 (0.61)	
**Original Discharge Summary Word Count**		
< 1000	5.2 (0.59)	0.938
1000-2000	5.2 (0.84)	
> 2000	5.3 (0.65)	
**Length of Stay**		
0-4 days	5.1 (0.77)	0.643
5-10 days	5.4 (0.63)	
>10 days	5.2 (0.71)	

Abbreviations: ANOVA, analysis of variance; SD, standard deviation

© 2024 Zaretsky J et al. *JAMA Network Open*.
