# Supplementary material for: Generative Artificial Intelligence to Transform Inpatient Discharge Summaries to Patient-Friendly Language and Format
Source: JAMA Netw Open. 2024 Mar 11;7(3):e240357. doi: 10.1001/jamanetworkopen.2024.0357 (PMC10928500; doi:10.1001/jamanetworkopen.2024.0357)
Supplement: Supplement 2. — Data Sharing Statement [file jamanetwopen-e240357-s002.pdf]

Data Sharing Statement

Zaretsky. Generative Artificial Intelligence to Transform Inpatient Discharge Summaries to Patient-Friendly Language and Format. *JAMA Netw Open*. Published March 11, 2024. doi:10.1001/jamanetworkopen.2024.0357

Data
Data available: No

Additional Information
Explanation for why data not available: This is for quality improvement purposes
